# Holmium Laser Lithotripsy in the New Stone Age: Dust or Bust?

**DOI:** 10.3389/fsurg.2017.00057

**Published:** 2017-09-29

**Authors:** Ali H. Aldoukhi, William W. Roberts, Timothy L. Hall, Khurshid R. Ghani

**Affiliations:** ^1^Division of Endourology, Department of Urology, University of Michigan, Ann Arbor, MI, United States; ^2^Department of Biomedical Engineering, University of Michigan, Ann Arbor, MI, United States

**Keywords:** ureteroscopy, lithotripsy, holmium laser, dusting, fragmentation

## Abstract

Modern day holmium laser systems for ureteroscopy (URS) provide users with a range of settings, namely pulse energy (PE), pulse frequency (Fr), and pulse width (PW). These variables allow the surgeon to choose different combinations that have specific effects on stone fragmentation during URS lithotripsy. Contact laser lithotripsy can be performed using fragmentation or dusting settings. Fragmentation employs settings of low Fr and high PE to break stones that are then extracted with retrieval devices. Dusting is the utilization of high Fr and low PE settings to break stones into submillimeter fragments for spontaneous passage without the need for basket retrieval. Use of the long PW mode during lithotripsy can reduce stone retropulsion and is increasingly available in new generation lasers. During non-contact laser lithotripsy, stone fragments are rapidly pulverized in a calyx in laser bursts that result in stones breaking into fine fragments. In this review, we discuss the effect of different holmium laser settings on stone fragmentation, and the clinical implications in a very much evolving field.

## Introduction

The prevalence of kidney stone disease has increased in the modern era due to the effects of increasing obesity, diabetes, and changes in dietary habits on urinary stone formation (1). In this new “Stone Age,” while minimally invasive therapies remain the mainstay of treatment, there has been a notable increase in the use of ureteroscopy (URS), so that URS is now the most common surgical therapy for upper urinary tract stones in North America (2, 3). One reason for the shift in practice toward URS has been the widespread availability of the holmium laser, which permits lithotripsy in all stone locations, regardless of stone composition. With the propagation of more powerful higher watt holmium systems and advances in laser technology, the options available to surgeons for laser settings and techniques to break up stones have expanded. In general, the surgical strategy for treating upper urinary tract stones with URS consists of either fragmentation and active basket retrieval or fragmentation resulting in fine fragments left *in situ* for spontaneous passage, also known as dusting technique. In the last few years, there have been several reviews detailing various advances in this field of endourology (4–7). In this article, we provide a synopsis of the different factors that should be considered when performing modern day holmium laser lithotripsy.

## Stone Fragmentation by the Holmium Laser

Energy emitted from the laser fiber following holmium laser activation leads to the formation of a vapor channel (cavitation bubble) through which the laser radiation is transmitted. The size of the bubble formed is directly proportional to the pulse energy (PE) and laser fiber size (8). A photothermal mechanism and chemical decomposition are the major contributing factors for stone fragmentation (9). The energy produced during bubble collapse (shock wave) has a limited role in stone fragmentation. Recently, advances in pulse modulation have resulted in the development of the “Moses technology” in which the laser emits part of the energy to create an initial bubble, and the remaining energy is discharged once the bubble is formed, so that it can pass through the already formed vapor channel (10). This new technology was adopted from the previously described phenomenon of holmium ablation—“Moses effect”—where the fluid is separated and a vapor channel is created (11).

## Contact Laser Lithotripsy

**Figure 1:**
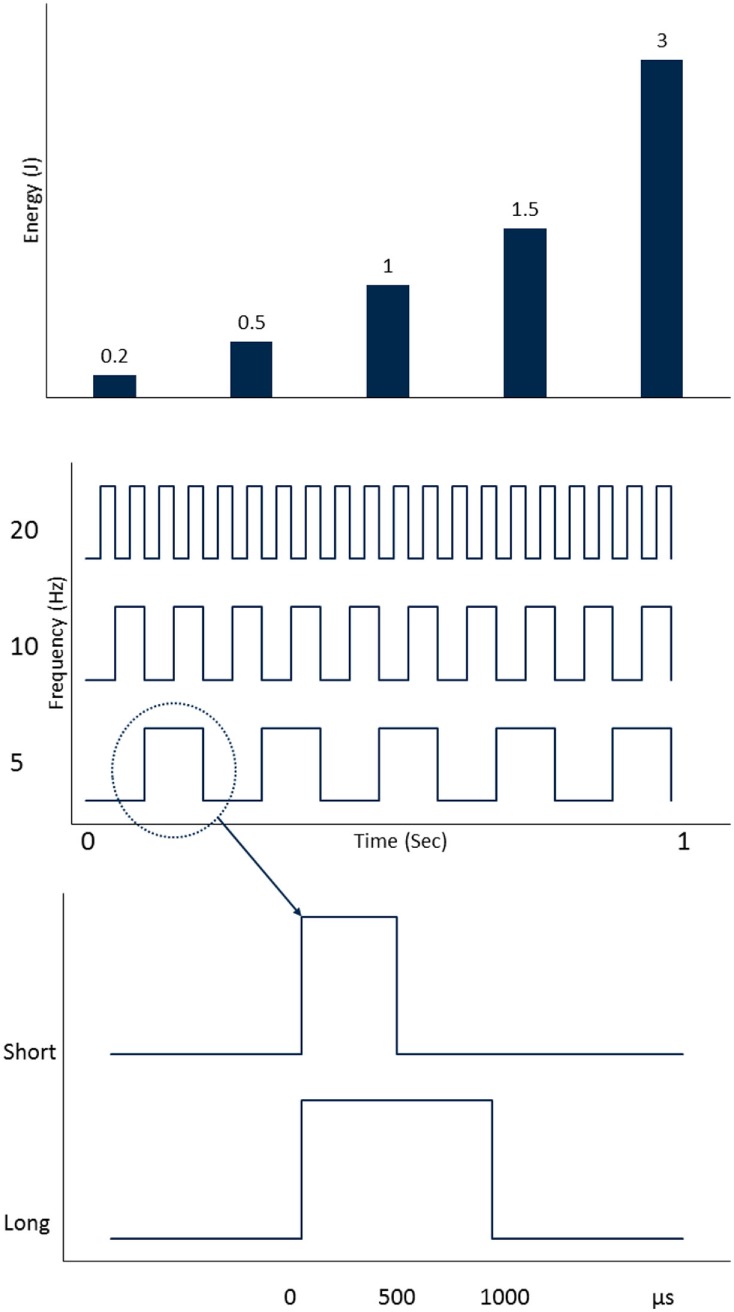
Holmium laser settings that are adjusted during laser lithotripsy (from top to bottom): pulse energy (PE), pulse frequency (Fr), and pulse width (PW).

The common step for both fragmentation and dusting strategies during URS is contact laser lithotripsy, where the stone is treated with the fiber touching the stone surface. Holmium lasers produce a thermal effect due to its strong absorption by water that causes stone vaporization. The amount of energy provided during lithotripsy depends on the PE and frequency (Fr) utilized; the total power (Watt) is a product of the PE (J) × Fr (Hz). The first generation of holmium lasers were low watt machines (≤20 W) and had limited PE and Fr ranges, with options for fragmentation restricted to low Fr and high PE (LoFr-HiPE). This resulted in classic fragmentation settings for lithotripsy such as 0.8–1.2 J × 4–10 Hz. The advent of multi-cavity high power holmium systems brought the ability to achieve low PE settings (<0.5 J) and high Fr’s (>20 Hz); the greater the power of the machine the higher the Fr possible (e.g., >50 Hz in 100 W systems). This lead to the development of a “Dusting” technique; commonly defined as laser lithotripsy utilizing high Fr and low PE (HiFr-LoPE) settings to break stones into fine (i.e., submillimeter) fragments. In a recent survey of Endourology Society members, 64% of urologists reported using these settings (12). More recently, holmium systems have incorporated the option to alter the pulse width (PW). Different combinations of PE, Fr, and PW during lithotripsy (Figure 1) permit different effects on stone fragmentation as well as having consequences on laser fiber efficacy (13).

### Pulse Energy

Holmium PE settings can range from 0.2 to 6.0 J depending on the power of the system. Traditionally, PE settings have been used at ranges between 0.6 and 1.2 J to fragment stones. The outcomes of altering PE on fragmentation have been reported in several studies; the higher the PE the greater the loss in stone mass (14–16). For instance, in a laboratory study by Kuo and colleagues, the percent loss in mass for a stone treated using a 200-µm laser fiber doubled and tripled when the PE was increased from 0.5 to 1.0 and 2.0 J, respectively (14). A similar observation was also noted in a study by Kronenberg and Traxer (17). Larger fragments are produced when high PE settings are used compared to when using lower PE settings (15). If performing a basket retrieval technique during URS, this result is desirable, so that 3- to 4-mm fragments can be extracted with a basket through the ureteral access sheath (UAS). A downside for increasing the PE and utilizing very high PE settings is retropulsion (13, 15). Consequences of retropulsion are migration of stone (such as from ureter to collecting system) and inefficient lithotripsy, thereby increasing procedural time. Lower PE settings result in smaller fragments breaking off the stone (15), which is more suited for a lithotripsy approach that relies on spontaneous passage and is preferred when using a dusting technique.

### Frequency

Frequency is defined as the number of pulses emitted from the laser fiber per second (Figure 1). Similar to PE, the range of pulse Fr’s available to the user depends on the power of the holmium system. Initial 15- to 20-W systems were limited to maximum frequencies of 15–20 Hz. Currently, holmium systems are able to achieve frequencies as high as 80 Hz. *In vitro* studies have shown that high Fr’s lead to more fragmentation at the same PE setting (14, 15, 17). Increasing the pulse Fr can also increase stone retropulsion, but not to the same degree as increasing the PE (15). In a recent laboratory study by Li et al., retropulsion force did not increase significantly when the pulse Fr was increased from 15 to 50 Hz (18). In an *in vitro* study examining various holmium laser settings incorporating optical coherence tomography to assess stone crater volumes, Sea et al. found that when using a constant amount of energy on a standardized stone model utilizing HiFr-LoPE (dusting) settings (0.2 J, 40 Hz), there were no fragments >1 mm in size (6). In contrast when using LoFr-HiPE settings (1.0 J, 10 Hz), the fragments were much larger. Smaller fragments are desired during dusting because they have a higher chance of spontaneous passage after URS. Higher Fr settings—which are now available in higher watt systems—have allowed a dusting technique to truly emerge, because the amount of time needed to break a stone utilizing low PE settings has been reduced.

### Pulse Width

Pulse width represents the time during which a single pulse is emitted from the laser, measured in microseconds (Figure 1). First-generation holmium systems operated in a single PW mode of approximately 350 µs. Recent systems have allowed the user to choose either short or long PW modes (range 500–1,500 µs). In a laboratory study assessing the effect of PW on stone fragmentation, the time needed to fragment an artificial stone using 1.0 J and 10 Hz was similar between short pulse (SP) and long pulse (LP) modes (19). However, when very high PE settings of 2.0 J were used, it took more time to fragment the stone using the SP mode. No significant differences in loss of stone mass between SP and LP modes were noted in a recent study by Wollin and coworkers (20). The main difference when utilizing PW is that LP results in less stone retropulsion (21–23). Kang and colleagues found that stones were displaced 30–50% more when SP was used compared to LP at comparable total power settings (22). A further advantage of the LP mode is its protective effect on laser fiber tip degradation, known as “burnback,” which can result in a reduction in the energy emitted from the fiber and a loss in its length. Fiber burnback increases when high PE settings are used and when using SP compared to LP mode (13, 21).

## Non-Contact Laser Lithotripsy

**Figure 2:**
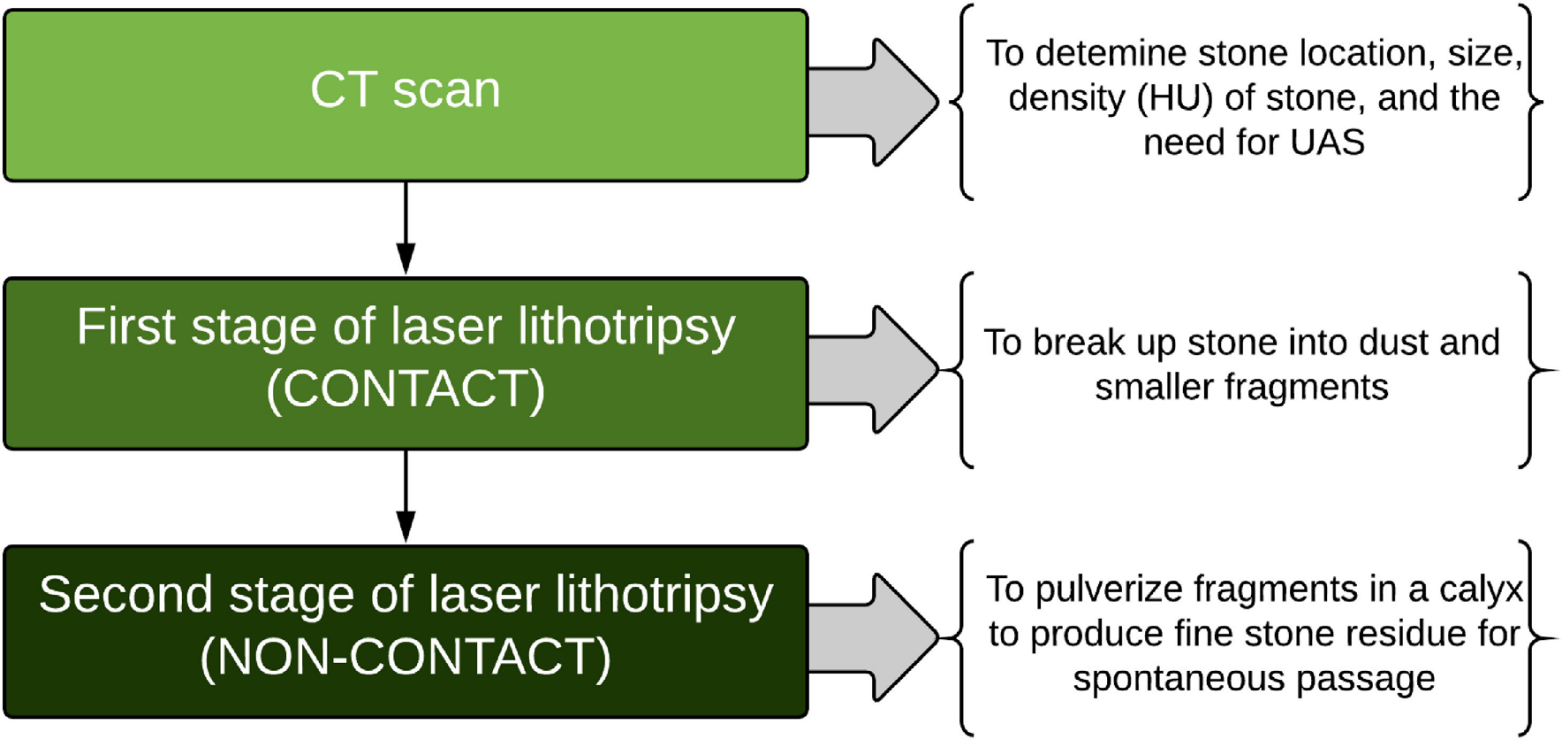
Surgical schema for treating upper urinary tract stones with dusting technique during ureteroscopic laser lithotripsy (HU: Hounsfield Unit; UAS: Ureteral Access Sheath).

In a dusting technique, after the stone is debulked resulting in numerous fragments, the next step is often non-contact laser lithotripsy. In this, stone fragments are pulverized in a calyx with the laser fiber activated in bursts, away from the stone fragments resulting in a whirlpool-like effect that causes stones to collide and fragment further. This is one hypothesized mechanism, and the other is laser vaporization of stone fragments as they swirl around. First described by Chawla et al., it is also commonly known as the “popcorn” effect, and settings for this have traditionally employed moderate-to-high PE and Fr (e.g., 1.0–1.5 J × 15–20 Hz) (24). In this initial report, a laser setting of 1.5 J and 40 Hz was reported as the most efficient for stone fragmentation, resulting in 63% loss of stone mass after 2 min of continuous laser firing (24). However, high PE’s may lead to significant fiber burnback. Recently, Emiliani and colleagues found that high PE (1.5 J) and high pulse Fr (40 Hz) resulted in more efficient popcorning. They also found longer lithotripsy time (4 vs 2 min) and smaller laser fiber (273 vs 365 µm) led to higher fragmentation success, which was defined as 50% reduction of stone volume (25). So far, the optimal settings that result in fine fragments, as well as the effects on fluid dynamics and temperature changes to the surrounding tissue are not fully understood. With the 120-W system, we have been utilizing a high Fr (50–80 Hz) popcorn technique utilizing a PE of 0.5 J, which we have called “pop-dusting.” This results in fine fragments without compromising fiber tip burnback. When evaluating patients with renal stones who underwent dusting with this system compared to patients treated with 60–100 W systems, the zero fragment stone clearance rate was significantly higher (26). Figure 2 describes our current schema for treating urinary stones using dusting technique during URS. Table 1 presents our current settings using the 120-W laser system.

**Table 1:** Dusting and fragmentation settings for ureteroscopy using 120 W (P120, Lumenis) holmium laser.

Stone location	Stone density	Fragmentation setting (J × Hz)	Dusting setting (J × Hz)
Ureter	Low density	0.8 × 6 (LP)	0.2 × 40 (LP)
	High density	1.2 × 6 (LP)	0.3 × 40 (LP)
Renal	Low density	1.0 × 6 (LP)	0.2 × 70 (LP)
	High density	1.4 × 6 (LP)	0.3 × 70 (LP)
Renal calyx	–	N/A	0.5 × 80 (SP) (pop-dusting)

*LP, long pulse; SP, short pulse*.

## Laser Fibers

In addition to the impact of different settings on stone fragmentation, fiber size can also affect the efficiency of laser lithotripsy. Differences between small (i.e., 200 µm) and larger fibers (i.e., 365 µm) on stone fragmentation have been noted when high PE settings (such as 2.0 J) have been used (14). Moreover, fiber size impacts the size of fragments produced during lithotripsy. Spore et al. found that 29% of fragments were >2 mm when a 365-µm fiber was used compared to 4% when a 272-µm fiber was used to fragment calcium oxalate monohydrate (COM) stones using a PE of 0.5 J (27). In addition, the size of the laser fiber influences scope flexibility and irrigation flow during URS, which is important if a dusting technique is utilized and optimal vision, is needed to see through the “snow storm” of fragments during lithotripsy (14).

Power output from laser fibers depend on the lasing time and cleaving method. The power output is highest when the fiber is new and has a smooth surface at the tip. It diminishes with time due to tip damage (28). For fiber cleaving, cleaving tools were found to be superior to scalpel and Mayo suture scissors by providing a higher power output initially (29). However, the power output from the fiber was found to be equivalent after a few minutes of laser firing regardless of the cleaving method or laser setting (28). It is unclear whether or not to strip a laser fiber prior to lithotripsy. Kronenberg and Traxer found that using unstripped, coated fibers provide better fragmentation than stripped fibers (30). In contrast, Ritchie and coworkers found that stripped fibers resulted in more efficient fragmentation compared to unstripped fibers due to better contact between the fiber tip and stone surface (31).

## Clinical Factors That Affect Laser Lithotripsy Settings

Stone location is a variable that should be considered when choosing technique and laser settings. Retropulsion is of greater concern when treating stones within the ureter. A fragmentation technique for a mobile ureteral stone might be a more efficient strategy, especially when ureteral fragments are easily retrieved with baskets. In contrast, when treating an impacted ureteral stone, it may be easier to break the stone initially utilizing dusting settings. However, one has to be careful regarding how much total power is applied in a ureter, with careful attention paid to keep lithotripsy targeting central to the stone, not peripheral, with use of high flow irrigation to limit excess heat generation in the confined spaces of the ureter.

Another factor that influences laser lithotripsy settings is the stone size. Stones that are large are much easier to treat with a dusting technique using painting and chipping methods during lithotripsy (32). However, dusting is not suitable for all stones, and *in vitro* studies have shown that crater volumes created during contact lithotripsy are dependent on the stone composition. At a low PE of 0.2 J, the volume of the crater for COM stones was significantly smaller than that achieved for uric acid and magnesium ammonium phosphate stones (15). With hard stones, higher PE is needed to obtain smaller fragments that can lead to fiber burnback and reduce lithotripsy efficiency. Also, the fragments may be sharp making spontaneous passage difficult. Assessing the stone density (Hounsfield Unit) on computed tomography (CT) may inform whether dusting is feasible, and if a UAS might be needed for basket retrieval.

## Clinical Studies Examining Dusting vs Retrieval

Clinical studies comparing dusting to retrieval techniques during URS are limited, with only one randomized trial so far (33). Schatloff and colleagues randomized patients with ureteral stones to either laser lithotripsy with intraoperative fragment retrieval (*n* = 30) or lithotripsy with spontaneous passage of fragments (*n* = 30). Utilizing an 80-W system, fragmentation settings of 0.8–1.0 J and 8–10 Hz were used, with patients in the spontaneous passage group undergoing exhaustive lithotripsy until fragments were dust or less than 2 mm (33). Stone sizes were equivalent between groups. The study found that the rate of emergency department (ED) visits within 30 days was significantly lower for patients that underwent retrieval (3%) compared to patients undergoing non-retrieval (30%). However, stone-free rates (SFRs) were not statistically different between the groups. This study was not performed utilizing HiFr-LoPE settings, and in general, there is a paucity of data on outcomes for patients undergoing lithotripsy utilizing such techniques (34). Furthermore, it is a misconception to consider that basket retrieval equals completely stone-free, as even in the hands of expert URS surgeons undertaking fastidious retrieval after URS, complete SFRs using CT follow-up approach only 55–60% (35, 36).

More recently, Chew and colleagues reported results from a prospective study of multiple centers where patients underwent URS laser lithotripsy with active retrieval or dusting (37). They found there were significantly more residual fragments in the dusting group, while complications and ED visits were not significantly different between the groups. However, this study has not yet been published, and it is not clear if patients in both groups were matched for stone sizes, and what settings were utilized for patients undergoing dusting. When trying to determine the superiority of dusting vs retrieval techniques, the lack of randomized studies utilizing CT to assess SFRs remains a major limitation in this arena (34). Each method has its own advantages and disadvantages (Table 2), and the decision as to which strategy is employed should be made based on the clinical scenario and the available resources (6).

**Table 2:** Advantages and disadvantages of dusting and retrieval techniques during ureteroscopic laser lithotripsy.

Method	Advantages	Disadvantages
Ureteroscopic stone dusting	Produces smaller fragments Avoid routine use of post-operative stenting Avoid routine use of ureteral access sheath (UAS) Shorter operation time No need for assistant	Utilizes high power laser system (high capital equipment cost) May not be suitable for hard stones (e.g., calcium oxalate monohydrate) Stone-free rate may depend on the surgeon skill Concern for fragment drainage in certain patients (e.g., spinal cord injury)
Ureteroscopy, fragmentation and basket retrieval	Uses low power laser system (low capital equipment cost) Ability to extract complete stone in non-complicated cases Suitable for hard stones	Produces larger fragments Longer operation time Higher disposable costs Need for assistant Risk of ureteral injury from using UAS Routine ureteral stenting if using sheath

## Conclusion

An understanding of holmium laser settings will permit the surgeon to utilize various techniques for URS lithotripsy. During contact laser lithotripsy, use of high PE settings leads to a greater loss in stone mass and is an important variable when using a fragmentation approach. Low PE settings result in smaller fragments, and along with high frequencies, is the foundation for a dusting technique resulting in submillimeter fragments. LP improves fragmentation efficiency by reducing retropulsion and may have a protective effect on laser fiber burnback. Non-contact laser lithotripsy is an end game strategy that can pulverize small stones in a calyx into fine fragments. However, not all stones are suitable for a dusting approach, and further clinical studies are needed to optimally define the role of these techniques during holmium laser lithotripsy.

## Author Contributions

AA and KG drafted the manuscript. WR, TH, and KG critically revised the manuscript. All the authors commented on and approved the final manuscript.

## Conflict of Interest Statement

KG is a consultant for Lumenis and Boston Scientific and has an investigator grant from Boston Scientific. All other authors declare that the research was conducted in the absence of any commercial or financial relationships that could be construed as a potential conflict of interest.
